# Acute akathisia with quetiapine: A case report and review of literature

**DOI:** 10.4103/0253-7613.71896

**Published:** 2010-12

**Authors:** Ruchita Shah, Sandeep Grover, Uma Maheshwari, Natasha Kate, Nidhi Malhotra

**Affiliations:** Department of Psychiatry, Postgraduate Institute of Medical Education and Research, Chandigarh 160 012, India

**Keywords:** Akathisia, side effects, quetiapine

## Abstract

Quetiapine is an atypical antipsychotic which has been shown to have greater relative affinity for 5-HT_2A_ receptors than for D_2_ receptors, due to which it is thought to lead to lower incidence of extrapyramidal symptoms (EPS). However, over the years literature in the form of case reports have accumulated which shows that quetiapine can lead to akathisia, especially in subjects prone to develop EPS. In this study, we report the case of a 22-year-old female who developed akathisia with quetiapine 150 mg/day, which subsided with reduction in dose. We have also reviewed the existing literature with respect to akathisia with quetiapine.

## Introduction

Quetiapine, an atypical antipsychotic, has low-to-moderate affinity for D_1_, D_2_, 5-HT_1A_, and 5-HT_2A_ receptors and moderate-to-high affinity for the α_1_- and α_2_-adrenergic receptors. Like other atypical antipsychotic, quetiapine has greater relative affinity for 5-HT_2A_ receptors than for D_2_ receptors. Due to this receptor profile, quetiapine has been claimed to cause less extrapyramidal symptoms (EPS). This claim has also been validated by large controlled trials which have shown that quetiapine is associated with placebo level EPS and akathisia.[1,2] Earlier, antipsychotic-induced akathisia was believed to be due to an inhibition of dopaminergic neurons, but now it is believed that multiple neurotransmitters are involved.[3]

In recent times, few case reports and case series have reported occurrence of akathisia, dystonia, and Parkinsonism with quetiapine; however, data are still sparse.[3– 5] To add to this literature, we report a case of a 22-year-old woman who developed akathisia while on low dose of quetiapine.

## Case Report

A, 22-year-old woman, was diagnosed as having paranoid schizophrenia (DSM-IV) since 2½ years on the basis of delusion of persecution, auditory hallucinations of commenting and commanding type, smiling to self, anger outbursts and violent behavior, social withdrawal, decreased sleep, and appetite with severe sociooccupational dysfunction. These symptoms were not associated with any affective symptoms, head injury, seizures, loss of consciousness, or signs and symptoms of infection.

She was initially treated with paliperidone (9–12 mg/day) with which she developed EPS without much improvement in psychopathology leading to inpatient treatment. In the inpatient unit, she was started on olanzapine (up to 20 mg/day), with which she showed improvement and was discharged. Later, despite good compliance with medication she had relapse of symptoms. In the follow-up, olanzapine was increased to 25 mg/day and trifluperazine (up to 10 mg/day) and trihexyphenidyl up to 4 mg/day were also added, but her clinical condition kept on worsening leading to re-hospitalization. In view of the relapse, it was decided to stop olanzapine and to treat the patient with risperidone and trifluperazine combination. While the cross taper was being done, she developed symptoms (fever, generalized rigidity, tremors, and tachycardia) suggestive of neuroleptic malignant syndrome (NMS), following which all antipsychotics were stopped.

After stopping antipsychotics, psychotic symptoms worsened and after 12 days of stopping all antipsychotic drugs, when all signs and symptoms suggestive of possible NMS were resolved, she was started on quetiapine 25 mg/day. Over the next 6 days, the dose of quetiapine was increased at the rate of 25 mg/day to –150 mg/day. Trihexyphenidyl was continued at 4 mg/day throughout this period. At quetiapine 150 mg/day, patients started complaining of subjective restlessness and expressed a constant wish to move about. The restlessness was worse at rest and was relieved by moving her legs while sitting or by walking. She was unable to sit still at one place and would constantly move her legs or walk about. She was rated on Barnes Akathisia Rating Scale (BAS) and on global clinical assessment for akathisia, she was rated as four, indicative of marked akathisia. Following this, the dose of quetiapine was reduced to 100 mg/day with which the subjective and objective signs of akathisia subsided and the score on BAS fell to zero after 5 days. In view of the continuation of psychotic symptoms, need for early response and tolerability issues patient was started on electroconvulsive therapy and quetiapine was tapered off. Later, she was started on clozapine which was gradually increased to 187.5 mg/day with which her positive symptoms resolved completely. There was no recurrence of akathisia or other EPS even after stopping trihexyphenidyl.

## Discussion

Of the three short-term efficacy trials of quetiapine, two have reported placebo level incidence of akathisia with quetiapine.[1,2] Only one short-term randomized controlled trial has reported higher rate of akathisia with quetiapine compared with placebo (4% of subjects on high dose of quetiapine compared to 1% subjects on placebo),[6] whereas short-term trials which have compared quetiapine with other antipsychotics have reported significantly lower incidence of akathisia with quetiapine compared to haloperidol.[7] Long-term clinical trials and effectiveness trials have also shown that quetiapine leads to lower rates of akathisia compared to haloperidol[8] and risperidone,[9] but others have reported no difference in the rate of akathisia among quetiapine, olanzapine, and risperidone.[10]

There are only few case reports which have described akathisia with quetiapine. Catalano *et al*.[3] reported two cases, receiving 25 mg and 50 mg of quetiapine for insomnia, who developed akathisia. Desarker and Sinha[4] reported a case of quetiapine-induced akathisia and dystonia in a young male receiving 400 mg/day of quetiapine, which subsided after change of drug to aripiprazole (10 mg/day). Bharadwaj and Grover[5] reported a case akathisia with quetiapine.

In the index case manifestation of akathisia started when the dose of quetiapine was increased to 150 mg/day and subsided with reduction in the dose of quetiapine. Patient was off antipsychotic for 12 days prior to starting quetiapine and throughout the period before starting quetiapine and while on quetiapine the subject was on the same dose of trihexyphenidyl. These findings suggest that the akathisia was not due to withdrawal of antipsychotic or trihexyphenidyl, but was a true side effect of quetiapine, because the akathisia subsided with reduction in the dose of quetiapine.

As is evident from the available case reports, one of the major risk factors for the development of akathisia with quetiapine is history of EPS with other antipsychotic and such patients can develop akathisia even with low dose of quetiapine. In such subjects, caution should be exercised while prescribing quetiapine and dose should be increased gradually and patients should be monitored closely.

In general, quetiapine is considered to have low propensity to cause EPS due to higher 5-HT2a/D2 receptor antagonism or a fast dissociation from D2.[1,2] However, with the emerging evidence, it is now understood that it is not the dopamine mechanism alone which is responsible for akathisia with quetiapine. It is now believed that akathisia occurs due to an inhibition of dopaminergic neurons that are located in the ventral tegmental area which have significant input from the noradrenergic and serotonergic systems. Hence, any medication that decreases the release of dopamine in the ventral tegmental area directly or through serotonergic or noradrenergic systems, can lead to akathisia.[3]

## References

[1] Buckley PF (2004). Efficacy of quetiapine for the treatment of schizophrenia: A combined analysis of three placebo-controlled trials. Curr Med Res Opin.

[2] Potkin SG, Gharabawi GM, Greenspan AJ, Mahmoud R, Kosik-Gonzalez C, Rupnow MF (2006). A double-blind comparison of risperidone, quetiapine and placebo in patients with schizophrenia experiencing an acute exacerbation requiring hospitalization. Schizophr Res.

[3] Catalano G, Grace JW, Catalano MC, Morales MJ, Cruse LM (2005). Acute akathisia associated with quetiapine use. Psychosomatics.

[4] Desarker P, Sinha VK (2006). Quetiapine-induced acute dystonia and akathisia. Aust N Z J Psychiatry.

[5] Bharadwaj R, Grover S (2008). Parkinsonism and akathisia with quetiapine: 3 case reports. J Clin Psychiatry.

[6] Small JG, Hirsch SR, Arvanitis LA, Miller BG, Link CG (1997). Quetiapine in patients with schizophrenia. A high- and low-dose double-blind comparison with placebo. Seroquel Study Group. Arch Gen Psychiatry.

[7] McIntyre RS, Brecher M, Paulsson B, Huizar K, Mullen J (2005). Quetiapine or haloperidol as monotherapy for bipolar mania--a 12-week, double-blind, randomised, parallel-group, placebo-controlled trial. Eur Neuropsychopharmacol.

[8] Glick ID, Marder SR (2005). Long-term maintenance therapy with quetiapine versus haloperidol decanoate in patients with schizophrenia or schizoaffective disorder. J Clin Psychiatry.

[9] Mintzer JE, Mullen JA, Sweitzer DE (2004). A comparison of extrapyramidal symptoms in older outpatients treated with quetiapine or risperidone. Curr Med Res Opin.

[10] McEvoy JP, Lieberman JA, Perkins DO, Hamer RM, Gu H, Lazarus A (2007). Efficacy and Tolerability of Olanzapine, Quetiapine, and Risperidone in the Treatment of Early Psychosis: A Randomized, Double-Blind 52-Week Comparison. Am J Psychiatry.

